# Characteristics of oseltamivir-resistant influenza A (H1N1) pdm09 virus during the 2013–2014 influenza season in Mainland China

**DOI:** 10.1186/s12985-015-0317-1

**Published:** 2015-06-24

**Authors:** Weijuan Huang, Xiyan Li, Yanhui Cheng, Minju Tan, Junfeng Guo, Hejiang Wei, Xiang Zhao, Yu Lan, Ning Xiao, Zhao Wang, Dayan Wang, Yuelong Shu

**Affiliations:** National Institute for Viral Disease Control and Prevention, China CDC, Key Laboratory for Medical Virology, National Health and Family Planning Commission, 155 Changbai Road, Changping District, Beijing, 102206, PR China

**Keywords:** Influenza A (H1N1) pdm09, 50 % Inhibitory concentration, Oseltamivir, Antiviral-resistant

## Abstract

**Background:**

In this study, we analyzed the characteristics of oseltamivir-resistant influenza A (H1N1) pdm09 virus isolated from patients in mainland China during the influenza season from September 2013 through March 2014, and provide guidance on which antiviral to be used for clinical treatment.

**Methods:**

The all viruses collected from September 1, 2013 through March 31, 2014 were obtained from the Chinese National Influenza Surveillance Network. A fluorescence-based assay was used to detect virus sensitivity to neuraminidase inhibitors (NAIs). The hemagglutinin (HA) and neuraminidase (NA) gene of the oseltamivir-resistant viruses were sequenced.

**Results:**

A total of 24 (2.14 %) influenza A (H1N1) pdm09 viruses that were resistant to oseltamivir were identified. These 24 viruses were isolated from 23 patients and no epidemiological link among them could be identified. Except for one virus with the H275H/Y mixture substitution, all the other 23 viruses had H275Y substitution in the NA protein. Sequence analysis revealed that the amino acid substitutions in the HA protein of influenza A (H1N1) pdm09 viruses with H275Y substitution isolated from mainland China were similar to the viruses from clustered cases reported in the United States, and the amino acid substitutions in the NA protein were similar to the viruses reported in Sapporo, Japan in 2013–2014. All of the oseltamivir-resistant viruses in mainland China and Japan possessed additional substitutions N386K, V241I and N369K in the NA protein, while most (>89 %) resistant-viruses from the United States during the same period possess V241I and N369K and did not have the N386K substitution. The N386K substitution was also exist in most sensitive viruses during the same period in mainland China. The amino acid substitutions in both HA and NA protein differed from the clustered cases from Australia reported in 2011 with additional substitutions. The drug-resistant influenza A(H1N1) pdm09 viruses were from patients without any known NAIs medication history prior to sampling.

**Conclusions:**

During the influenza season from September 2013 through March 2014 in Mainland China, oseltamivir-resistant influenza A(H1N1)pdm09 viruses were much more frequently detected than ever since the appearance of the virus in 2009.

**Electronic supplementary material:**

The online version of this article (doi:10.1186/s12985-015-0317-1) contains supplementary material, which is available to authorized users.

## Background

The pandemic of 2009 highlighted the importance of global influenza viral surveillance for the detection of new virus variants and the need of antiviral medications to mitigate the public health impact of influenza. Oseltamivir is a drug commonly used for the prevention and treatment of influenza. During the 2009 influenza pandemic, oseltamivir was used worldwide and has been listed as a stockpiled drug in many countries in response to influenza pandemics [1, 2].

After pandemic of 2009, influenza A (H1N1) pdm09 (abbreviated as H1N1pdm09 here after) viruses became one of the seasonal influenza viruses. Prior to 2013, less than 1 % of H1N1pdm09 viruses worldwide were oseltamivir-resistant and most came from patients who had received oseltamivir treatment before specimen collection [3]. Most oseltamivir-resistant H1N1pdm09 viruses possessed histidine (H) to tyrosine (Y) change at amino acid position 275 of the NA genes [4]. From November 2013 through February 2014, a cluster of H1N1pdm09 viruses with H275Y substitution were detected in Sapporo, Japan. No epidemiological link were identified among the patients except for one family infection, and almost all of the patients had no exposure to NAIs before specimen collection [5], leading to concerns about local epidemics of oseltamivir-resistant viruses. In mainland China, the routine antiviral susceptibility surveillance to influenza virus with phenotypic method was established in 2010. During the influenza season from September 2013 through March 2014 in Mainland China, oseltamivir-resistant H1N1pdm09 viruses were much more frequently detected than ever since the appearance of the virus in 2009, and here we report the findings to provide data for global surveillance of antiviral-resistant influenza virus and guidance in the choice of antiviral drugs for clinical treatment.

## Results

### Neuraminidase inhibition (NI) assay result

During the 2013–2014 influenza season, H1N1pdm09 virus, A (H3N2) virus and B virus were co-circulation in Mainland China. In the NI assay, 1123 H1N1pdm09, 558 A (H3N2), and 918 influenza B viruses were tested for susceptibility to oseltamivir and zanamivir. Twenty-four H1N1pdm09 viruses exhibited more than 200-fold elevated 50 % inhibitory concentration (IC_50_, the concentration of drug required to inhibit a standardized amount of NA activity by 50 %) for oseltamivir compared to the mean IC_50_ of oseltamivir sensitive reference virus A/California/07/2009 (275H), which was used as a run control and can provide enough data points to calculate a mean IC_50_. The median IC_50_ of oseltamivir for these 24 viruses was 251.68 nM and ranged from 64.85 nM to 478.65 nM, while the median IC_50_ of zanamivir for these viruses was 0.21 nM, ranging from 0.16 nM to 0.41 nM (Table 1). Accord to the WHO AVWG (antiviral susceptibly expert working group) criteria [6], the above results were interpreted as highly reduced inhibition by oseltamivir but normal inhibition by zanamivir.

**Table 1:** The information of H1N1pdm09 oseltamivir-resistant viruses isolated during the 2013–2014 influenza season in Mainland China

Isolate name	Collection date^a^	Passage history^b^	Age (Year)/Sex^c^	IC_50_ (nM)		Neuraminidase inhibitor exposure history
				Oseltamivir	Zanamivir	
A/Beijing-Xichengnanpian/SWL11619/2013	2013-09-29	C2 + C1	27/F	242.80	0.18	No
A/Sichuan-Wuhou/SWL2259/2013	2013-09-30	E1 + E1	29/F	348.55	0.17	No
A/Chongqing-Yuzhong/SWL11434/2013	2013-10-12	E1 + E1	3/F	64.85	0.25	Unknown
A/Sichuan-Qingyang/SWL1599/2013	2013-11-03	C1 + C1	7/M	283.80	0.21	No
A/Hubei-Wuchang/SWL1322/2013	2013-11-09	C2 + C1	30/F	257.90	0.22	Unknown
A/Chongqing-Banan/SWL1690/2013	2013-11-11	C2 + C1	15/F	176.60	0.23	No
A/Chongqing-Banan/SWL1732/2013	2013-11-21	E2 + E1	7/F	237.50	0.30	No
A/Hunan-Furong/SWL1543/2013	2013-12-02	C2 + C1	51/F	277.85	0.20	No
A/Fujian-Gulou/SWL11609/2013	2013-12-03	E1 + E1	27/F	401.15	0.27	Unknown
A/Shanghai-Changning/SWL1621/2013	2013-12-03	E3 + E1	63/F	269.60	0.32	No
A/Shanghai-Changning/SWL1621/2013		C1 + C1		210.65	0.27	
A/Chongqing-Banan/SWL1804/2013	2013-12-03	C1 + C1	71/M	287.80	0.21	No
A/Chongqing-Fuling/SWL1638/2013	2013-12-05	C2 + C1	15/M	245.45	0.17	No
A/Hubei-Xiangcheng/SWL1750/2013	2013-12-05	C1 + C1	1/F	187.90	0.17	No
A/Guangxi-Gangbei/SWL1481/2013	2013-12-17	C1 + C1	14/M	272.25	0.20	No
A/Heilongjiang-Daoli/SWL1480/2013	2013-12-17	C3 + C1	9/M	148.11	0.21	Unknown
A/Heilongjiang-Saertu/SWL1655/2013	2013-12-18	C2 + C1	17/M	121.47	0.20	Unknown
A/Guangdong-Liwan/SWL1865/2013	2013-12-18	C2 + C1	24/M	244.30	0.19	No
A/Hubei-Fancheng/SWL210/2014	2014-01-05	C1 + C1	19/M	301.55	0.21	No
A/Jilin-Longshan/SWL117/2014	2014-01-07	C1 + C1	39/M	216.75	0.18	No
A/Guizhou-Nanming/SWL1108/2014	2014-01-08	C1 + C1	3/M	190.40	0.16	Unknown
A/Hunan-Tianyuan/SWL129/2014	2014-01-08	C1 + C1	3/F	298.60	0.24	No
A/Neimenggu-Yuquan/SWL1155/2014	2014-02-27	E2 + E1	38/M	262.75	0.41	No
A/Jiangxi-Yushui/SWL1220/2014	2014-03-12	C2 + C1	1/M	478.65	0.37	No
A/Califorina/07/2009(275H)				0.30	0.15	
A/North Carolina/39/2009(H275Y)				201.27	0.18	

^a^Collection date: Collection date of clinical specimen

^b^C: MDCK; E: embryonated egg. Number is passage number in cell/eggs

^c^F: Female; M: Male

### Epidemiological characteristics of oseltamivir-resistant viruses

These 24 viruses with highly reduced susceptibility to oseltamivir were distributed throughout the influenza season with varied proportions (Table 2), and most of the oseltamivir-resistant viruses were isolated from clinical specimens collected in December 2013 (Table 2).

**Table 2:** The time distribution of H1N1pdm09 virus with highly reduced sensitivity to oseltamivir during the 2013–2014 influenza season in Mainland China

Sample collection time	No. of viruses tested	No. of viruses with highly reduced sensitivity to Oseltamivir (%)
Sep-2013	35	2 (5.71 %)
Oct-2013	32	1 (3.13 %)
Nov-2013	113	4 (3.54 %)
Dec-2013	226	11 (4.87 %)
Jan-2014	150	4 (2.67 %)
Feb-2014	345	1 (0.29 %)
Mar-2014	222	1 (0.45 %)
Total	1123	24 (2.14 %)

The 24 viruses were actually collected from 23 patients, as two viruses, A/Shanghai-Changning/SWL1621/2013 egg isolate and A/Shanghai-Changning /SWL1621/2013 Madin-Darby canine kidney (MDCK) cell isolate were isolated from the same sample. All samples were collected from the hospital outpatients, including 11 females and 12 males. The median age was 19 years, including 5 children between 1 and 6 years old; the maximum age was 71 years, and the minimum age was 1 year. All 23 patients with NA H275Y virus had no history of exposure to NAIs before sample collection except 6 with unknown history (Table 1). Three cases were from the same district of Chongqing province, the rest were from different regions of 14 provinces and municipalities in northern and southern China, and all were sporadic cases without known epidemiological relationship. The collecting dates of the 3 viruses from the same district were approximately 10 days apart from each other and the 3 patients had no common epidemiological exposure history.

### Phylogeny analysis of NA and HA genes from oseltamivir-resistant viruses

**Fig. 1:**
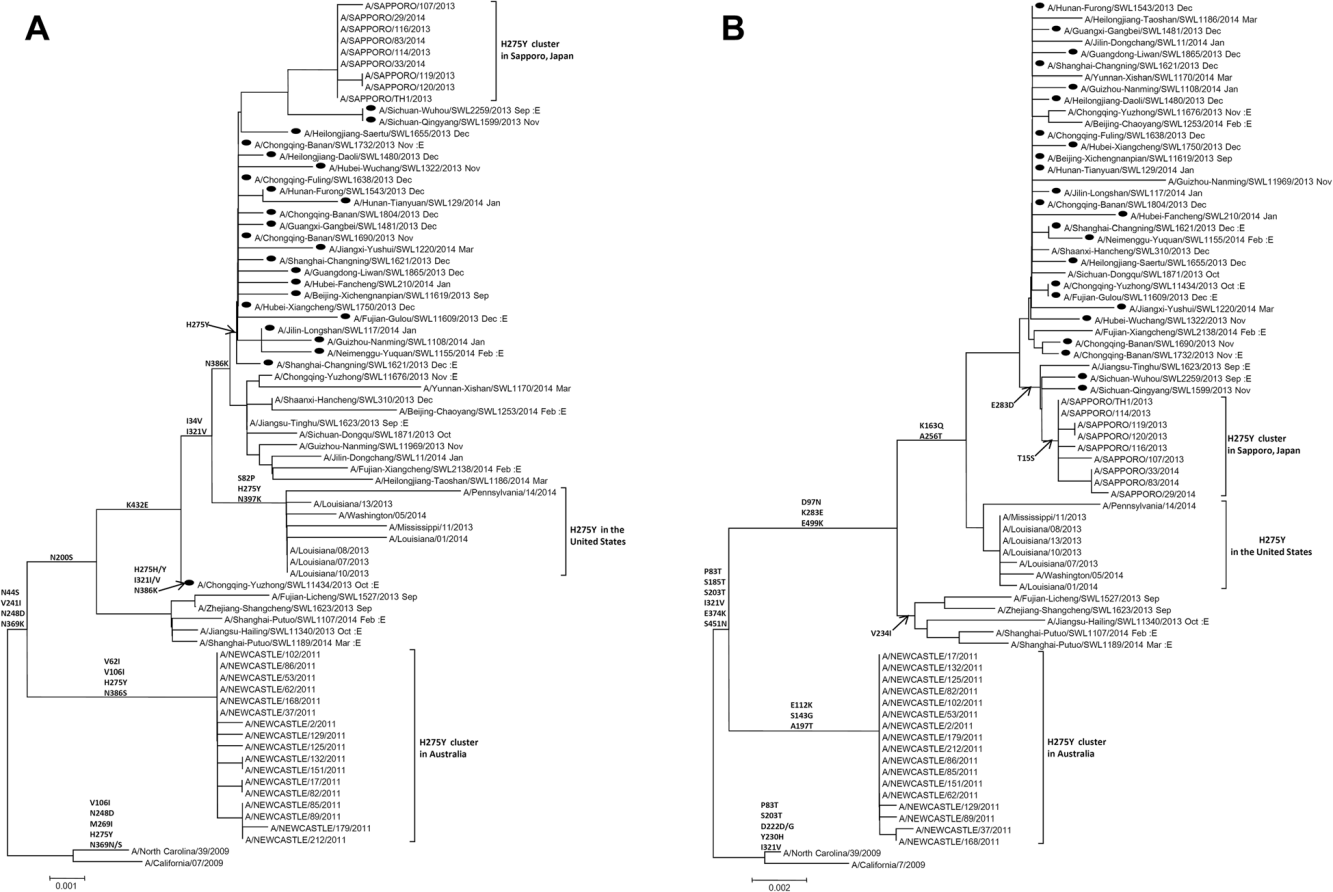
Phylogenetic analysis of the Neuraminidase (**a**) and hemagglutinin (**b**) genes of the H275Y mutant H1N1pdm09 Viruses isolated in mainland China in 2013–2014 influenza season. The clustered H1N1pdm09 virus with H275Y mutation in NA gene reported in Japan in 2013–2014, the United States in 2013–2014, and Australia in 2011 were indicated with a vertical line. The NA gene of the H275Y mutant H1N1pdm09 Viruses isolated in mainland China in 2013–2014 influenza season were indicated Solid circle. A/ California /07/2009: reference virus with 275H; A/North Carolina/ 39/2009: reference virus with H275Y. E: Embryonated egg isolate

Representative H1N1pdm09 viruses isolated in mainland China during the 2013–2014 influenza season were analyzed phylogenetically, and in together with H275Y mutant H1N1pdm09 viruses detected in Sapporo, Japan [5] and United States [7] in 2013–2014 and in the Australia cluster in 2011 [8]. Phylogenetic analysis of NA (Fig. 1a) and HA (Fig. 1b) gene sequences showed, in both phylogenetic trees, that the oseltamivir-resistant viruses did not emerge from a common source. However, the oseltamivir-resistant viruses from Mainland China were genetically similar to those from the Sapporo cluster. The H1N1pdm09 viruses from mainland China collected during the 2013–2014 influenza season belonged to the same clade, and oseltamivir-resistant viruses were generally scattered among the sensitive viruses.

### Genetic characteristics of oseltamivir-resistant viruses

Twenty three of the 24 viruses possessed H275Y substitution, while A/Chongqing-Yuzhong/SWL11434/2013 possessed H275H/Y mixture substitution. As substitutions in the HA and NA protein may compensate the destabilizing effect of H275Y substitution [9], we further characterized the other amino acid substitutions in the HA and NA protein of the oseltamivir-resistant viruses (Tables 3 and 4).

**Table 3:**
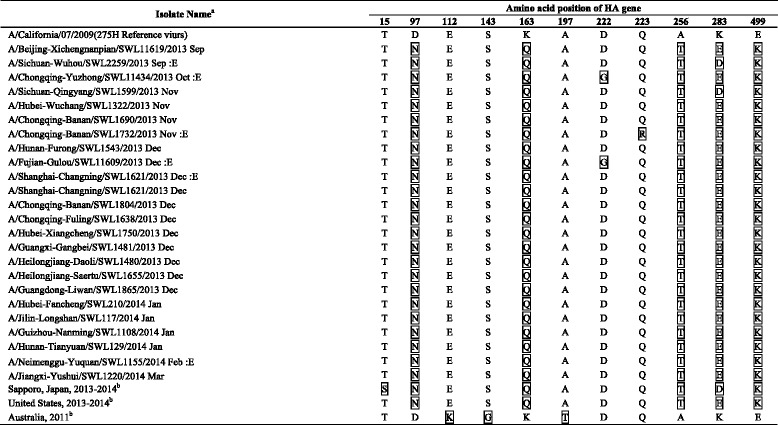
Characteristic of amino acids of the HA gene of oseltamivir-resistant H1N1pdm09 viruses isolated in mainland China in 2013–2014 influenza season

The boxed amino acids: Different amino acid substitutions of the viruses in comparison with A/California/07/2009

^a^: E: Embryonated egg isolate

^b^: Consensus sequence of the viruses from related clustered cases

**Table 4:**
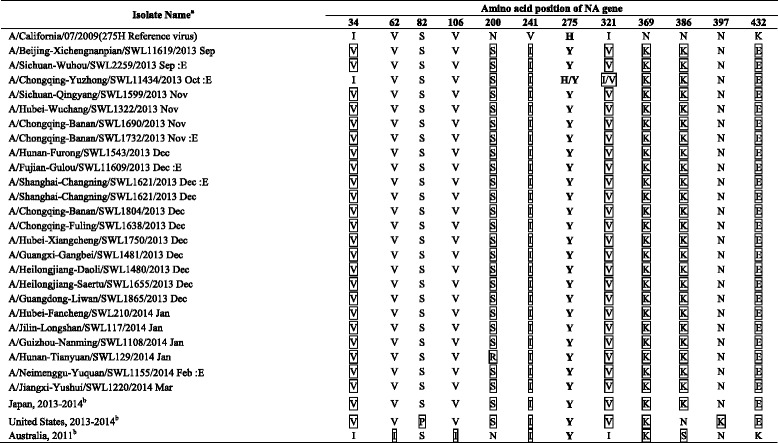
Characteristic of amino acids of the NA gene of oseltamivir-resistant H1N1pdm09 viruses isolated in mainland China in 2013–2014 influenza season

The boxed amino acids: Different amino acid substitution of the viruses in comparison with A/California/07/2009

^a^E: Embryonated egg isolate

^b^: Consensus sequence of the viruses from related clustered cases

These substitutions in the HA protein of the oseltamivir-resistant viruses isolated from Mainland China were in consistent with those of the United States viruses, but more distinct from those of the Australian viruses, and had one amino acid substitution (position 15) different from those from the Sapporo cluster. In addition, among all oseltamivir-resistant viruses from Mainland China, A/Chongqing-Yuzhong/SWL 11434/2013 and A/Fujian-Gulou/SWL11609/2013 had D222G amino acid substitutions, and A/Chongqing-Banan/SWL 1732/2013 had Q223R amino acid substitutions in the HA protein. D222G and Q223R substitutions in the HA protein of H1N1pdm09 viruses were known to be associated with increased pathogenicity of the viruses [10–12]. However, all samples were collected from the outpatients with mild disease and there was no severe case involved. For the NA gene, the amino acid of oseltamivir-resistant viruses isolated from mainland China were consistent with those of the Japanese viruses, and more distinct with that of Australian viruses. All of the oseltamivir-resistant viruses from mainland China and the Sapporo cluster possessed an additional N386K substitution, besides two amino acid substitutions, V241I and N369K, in the NA protein, which might compensate for the detrimental effect of the H275Y substitution on viral fitness [3]. The N386K substitution also occurred in most sensitive viruses during in the same period in mainland China. No specific HA gene mutation that may has a compensatory role in replication of an oseltamivir-resistant influenza virus was identified, comparing with the sensitive viruses isolated from the same period in mainland China.

## Discussion

From September 2013 through March 2014, 24 H1N1pdm09 viruses with highly reduced sensitivity to oseltamivir were detected in mainland China. The proportion of resistant viruses was 2.14 % with highest proportion of 5.71 % in September, which is higher than the 1 % proportion worldwide in the previous year [3], and similar to the proportion of 2 % for the circulating viruses tested during the 2013–2014 influenza season globally [13]. Except for H275Y substitution in the NA protein, no other substitution previously reported to be related to reduced susceptibility to oseltamivir was detected in the 24 resistant viruses.

One limitation of the study is the lack of sequences obtained from corresponding clinical specimens, because only seasonal influenza virus isolates were submitted to Chinese National Influenza Center and clinical specimens were not available in the routine surveillance.

In terms of HA gene mutation, the H1N1pdm09 viruses with H275Y substitution isolated from mainland China were more similar to the clustered cases from the United States in 2013–2014. However, with regard to the NA gene phylogenetic tree, the H1N1pdm09 viruses isolated from mainland China were more similar to those from Japan, in 2013–2014. Thus, it is possible that the NA genes of the H1N1pdm09 viruses isolated from mainland China and Japan shared the same origin. The 24 viruses were isolated from 23 cases from 14 provinces or municipalities in mainland China with the first case detected in September 2013 in China. The first clustered case in Sapporo, Japan, was detected in November 2013. Therefore, it is plausible that this virus was introduced to Japan from China, and further mutation in the HA and internal genes of the Japanese oseltamivir resistant H1N1pdm09 viruses might enable this virus to have caused a limited human-to-human transmission in Sapporo, Japan and distinct from the sporadic H275Y mutant viruses in Japan during the same period [5].

The seasonal influenza A (H1N1) viruses, which contain an H275Y substitution in the NA protein, were first detected in the Norway and subsequently elsewhere in Europe in the early 2008, and then spread globally within nine months, indicating that other amino acid substitutions may have compensate the destabilizing effect from H275Y substitutions, thus enabling the spread of viruses with the H275Y substitutions in the population [9, 14]. After the H1N1pdm09 pandemic started, a widespread community cluster of an oseltamivir-resistant H1N1pdm09 virus occurred in 2011 in Newcastle, Australia [8]. This resistant virus possessed the H275Y substitution and three additional substitutions, V241, N369K and N386S, in the NA protein. The V241I and N369K substitutions, were reported to confer robust viral fitness on the H275Y mutant virus [15, 16]. The vast majority of recently circulating H1N1pdm09 viruses possessed two amino acid substitutions, V241I and N369K, in the NA protein [3]. The N386S substitution, however, decreased the enzymatic activity and surface expression of NA in infected cell, suggesting a negative effect on virus fitness [15]. Similarly, the 24 viruses from mainland China and the viruses from Sapporo, Japan possessed the V241I, N369K and N386K substitutions. However, most (>89 %) resistant-viruses from the United States during the same period did not have the N386K substitution [7]. Before the 2013–2014 influenza season, H275Y mutant virus with V241I, N369K and N386K substitutions was not detected in Mainland China, or Japan [17]. It was reported the N386K destabilizes the NA structure in the presence of the V241I and N369K substitutions, causing a negative effect on virus fitness [5]. Therefore, the structure of the mutant NA molecule was less stable than that of the sensitive virus, presumably because of the N386K substitution. In addition to permissive NA mutations, other properties which might provide an advantage to oseltamivir-resistant viruses and facilitate their spread, should also be monitored.

Oseltamivir was widely used for influenza prevention and treatment in 2009, and has been listed as a stockpiled drug in response to influenza pandemics in many countries after the 2009 pandemic. Emergence of the clustered cases in Australia in 2011, in the United States and Japan in 2013–2014, as well as the rise in the proportion of oseltamivir-resistant H1N1pdm09 virus in Mainland China from September 2013 through March 2014, with most of the patients weren’t being treated with NAIs before sampling, has indicated that the human-to-human transmission capacity of the H1N1 pdm09 viruses with H275Y substitution is gradually increasing. Fortunately, in our study, the H1N1pdm09 viruses with H275Y substitution in NA protein were still sensitive to zanamivir. Therefore, in addition to strengthening the enhanced surveillance of antiviral susceptibility of influenza virus, we should pay attention to clinical drug choices, development of new drugs, and strive to improve capabilities for influenza prevention and control.

## Conclusions

During the 2013–2014 influenza season, prevalence of the oseltamivir-resistant H1N1pdm09 viruses were much more frequently detected than ever since the appearance of the virus in 2009 in Mainland China. Most patients infected with an oseltamivir-resistant H1N1pdm09 virus had no prior exposure to oseltamivir. Our results emphasize the need for surveillance for neuraminidase inhibitor susceptibility among circulating influenza viruses.

## Materials and methods

### Viruses

H1N1pdm09 viruses isolated from specimens collected from September 1, 2013 through March 31, 2014 were obtained from the Chinese National Influenza Surveillance Network, which covers 32 provinces in mainland China (including the autonomous regions/municipalities directly under the Central Government), including 408 network laboratories and 554 sentinel hospitals. The viruses were submitted to Chinese National Influenza Center and propagated in either MDCK cells or embryonated chicken eggs prior for testing. In addition, reference H1N1pdm09 virus variant, A/North Carolina/39/2009 which carrying known marker (275Y) of highly reduced susceptibility in the NI assays and the drug-susceptible wild type counterparts, A/California/07/2009 (275H) were used in this study. The National Health and Family Planning Commission deemed the data collection for this surveillance to be part of the continuing public health investigation and exempt from institutional review board assessment.

### Neuraminidase inhibition (NI) assay

The Chinese National Influenza Center used a fluorescence-based assay, NA-Fluor™ kit (Applied Biosystems, Foster City, CA, USA) to determine virus sensitivity to NAIs as reported [18, 19]. Oseltamivir carboxylate was provided by Hoffman-La Roche (Basel, Switzerland), zanamivir was provided by Glaxo Smith Kline (Uxbrige, UK), and Fluorescent was measured using 2104 multilabel reader (Envision™, Perkin Elmer, USA) with the excitation and emission wavelengths of 355 and 450 nm.

### IC_50_ analysis

IC_50_ values were determined with the GraphPad Prism 5 software (GraphPad Software, La Jolla, CA, USA). Interpretation of IC_50_ was performed using the WHO AVWG criteria: the testing virus was compared with the drug-sensitive reference virus, for influenza A viruses, a < 10-fold increase in IC_50_ represents normal inhibition, and a 10–-100 fold increase represents reduced inhibition, while a > 100-fold increase is highly reduced inhibition [6].

### HA and NA gene sequencing

Virus with an IC_50_ of ≥10-fold, compared to that of the drug-sensitive reference virus, were analyzed by HA and NA gene sequencing. Bio Robot M48 automated nucleic acid extraction instrument (Qiagen, Germany) and its compatible MagAttract Viral RNA M48 kit (96) (Qiagen, Germany) were used for viral RNA extraction. One Step reverse transcription-polymerase chain reaction (RT-PCR) kit (Qiagen, Germany) was used for nucleotide sequences amplification and the PCR reaction conditions were as follows: 60 °C for 1 min; 42 °C for 20 min; 50 °C for 20 min; 95 °C for 15 min; then 94 °C for 30 s, 55 °C for 30 s, and 72 °C for 1 min for a total of 35 cycles; followed by final extension at 72 °C for 10 min. PCR primers for amplification of HA and NA genes were obtained from the JCVI Genomic Sequencing Center for Infectious Diseases website (http://gsc.jcvi.org/projects/msc/influenza/). PCR products were purified using the QIAquick 96 PCR Purification kit (24) (Qiagen, Germany), followed by sequencing reactions using BigDye® Terminator v3.1 Cycle Sequencing Kit (Applied Biosystems, Foster, USA). The sequencing primers were M13F: 5′-TGTAAAACGACGGCCAGT-3′ and M13R: 5′-CAGGAAACAGCTATG ACC-3′. The sequencing reaction conditions were 96 °C for 1 min; 25 cycles of 96 °C for 10 s, 50 °C for 5 s, and 60 °C for 4 min. After sequencing products were purified using the Bigdye® XTerminator™ Purification kit (Applied Biosystems, Foster, USA), sequences were read by 3730XL DNA sequencer (Applied Biosystems, Foster, USA).

### Sequence analysis

Sequences were assembled using the Seqman of the Lasergene package (DNAStar, Corporation, USA). Nucleotide alignments and phylogenetic tree were constructed with MEGA version 5.0 software (the Biodesign Institute, USA) using the neighbor-joining (NJ) method, and 1000 bootstrap replications were performed to evaluate the reliabilities. The H1N1pdm09 viruses from community in Australia, Japan, and the United States, with H275Y in NA gene, were analyzed in together with the Chinese viruses, with sequences from the Global Initiative on Sharing Avian Influenza Data (GISAID) [5, 7, 8]. The accession number are present in the Additional file 1: Table S1. Amino acids are described with the N1 numbering.

## Consent

Written informed consent was obtained from the patient for the publication of this report and any accompanying images.

## Additional file

Additional file 1: Table S1. The GISAID accession numbers of the virus genes used in the phylogenetic trees.

## References

[1] Ling LM, Chow AL, Lye DC, Tan AS, Krishnan P, Cui L (2010). Effects of early oseltamivir therapy on viral shedding in 2009 pandemic influenza A (H1N1) virus infection. Clin Infect Dis.

[2] Campbell CN, Mytton OT, McLean EM, Rutter PD, Pebody RG, Sachedina N (2010). Hospitalization in two waves of pandemic influenza A(H1N1) in England. Epidemiol Infect.

[3] Meijer A, Rebelo-de-Andrade H, Correia V, Besselaar T, Drager-Dayal R, Fry A (2014). Global update on the susceptibility of human influenza viruses to neuraminidase inhibitors, 2012–2013. Antiviral Res.

[4] Gubareva LV, Kaiser L, Hayden FG (2000). Influenza virus neuraminidase inhibitors. Lancet.

[5] Takashita E, Kiso M, Fujisaki S, Yokoyama M, Nakamura K, Shirakura M (2015). Characterization of a large cluster of influenza A(H1N1)pdm09 virus cross-resistant to oseltamivir and peramivir during the 2013/2014 influenza season in Japan. Antimicrob Agents Chemother..

[6] WHO/Meetings of the WHO working group on surveillance of influenza antiviral susceptibility-Geneva. November 2011and June 2012. Wkly Epidemiol Rec (WER). 2012; 87: 369–374. 23061103

[7] Okomo-Adhiambo M, Fry AM, Su S, Nguyen HT, Elal AA, Negron E (2015). Oseltamivir-resistant influenza A(H1N1)pdm09 viruses, United States, 2013–14. Emerg Infect Dis.

[8] Hurt AC, Hardie K, Wilson NJ, Deng YM, Osbourn M, Leang SK (2012). Characteristics of a widespread community cluster of H275Y oseltamivir-resistant A(H1N1)pdm09 influenza in Australia. J Infect Dis.

[9] Bloom JD, Gong LI, Baltimore D (2010). Permissive secondary mutations enable the evolution of influenza oseltamivir resistance. Science.

[10] Chutinimitkul S, Herfst S, Steel J, Lowen AC, Ye J, van Riel D (2010). Virulence-associated substitution D222G in the hemagglutinin of 2009 pandemic influenza A(H1N1) virus affects receptor binding. J Virol.

[11] Liu Y, Childs RA, Matrosovich T, Wharton S, Palma AS, Chai W (2010). Altered receptor specificity and cell tropism of D222G hemagglutinin mutants isolated from fatal cases of pandemic A(H1N1) 2009 influenza virus. J Virol.

[12] Zhang Y, Zhang Q, Gao Y, He X, Kong H, Jiang Y (2012). Keymolecular factors in hemagglutinin and PB2 contribute to efficient transmission of the 2009 H1N1 pandemic influenza virus. J Virol.

[13] Takashita E, Meijer A, Lackenby A, Gubareva L, Rebelo-de-Andrade H, Besselaar T (2015). Global update on the susceptibility of human influenza viruses to neuraminidase inhibitors, 2013–2014. Antiviral Res.

[14] Ginting TE, Shinya K, Kyan Y, Makino A, Matsumoto N, Kaneda S (2012). Amino acid changes in hemagglutinin contribute to the replication of oseltamivir-resistant H1N1 influenza viruses. J Virol.

[15] Butler J, Hooper KA, Petrie S, Lee R, Maurer-Stroh S, Reh L (2014). Estimating the fitness advantage conferred by permissive neuraminidase mutations in recent oseltamivir-resistant A(H1N1)pdm09 influenza viruses. PLoS Pathog.

[16] Abed Y, Pizzorno A, Bouhy X, Rhéaume C, Boivin G (2014). Impact of potential permissive neuraminidase mutations on viral fitness of the H275Y oseltamivir-resistant influenza A(H1N1)pdm09 virus in vitro, in mice and in ferrets. J Virol.

[17] Takashita E, Ejima M, Itoh R, Miura M, Ohnishi A, Nishimura H (2014). A community cluster of influenza A(H1N1)pdm09 virus exhibiting cross-resistance to oseltamivir and peramivir in Japan, November to December 2013. Euro Surveill.

[18] Nguyen HT, Sheu TG, Mishin VP, Klimov AI, Gubareva LV (2010). Assessment of pandemic and seasonal influenza A(H1N1) virus susceptibility to neuraminidase inhibitors in three enzyme activity inhibition assays. Antimicrob Agents Chemother.

[19] Okomo-Adhiambo M, Sleeman K, Ballenger K, Nguyen HT, Mishin VP, Sheu TG (2010). Neuraminidase inhibitor susceptibility testing in human influenza viruses: a laboratory surveillance perspective. Viruses.

