# Supplementary material for: Characteristics of oseltamivir-resistant influenza A (H1N1) pdm09 virus during the 2013–2014 influenza season in Mainland China
Source: Virol J. 2015 Jun 24;12:96. doi: 10.1186/s12985-015-0317-1 (PMC4484626; doi:10.1186/s12985-015-0317-1)
Supplement: Additional file 1: Table S1. — The GISAID accession numbers of the virus genes used in the phylogenetic trees. [file 12985_2015_317_MOESM1_ESM.docx]

**Additional file 1: Table S1 The GISAID accession numbers of the virus genes used in the phylogenetic trees**

| **The accession number of HA gene** | **The accession number of NA gene** | **Isolate name** |
| --- | --- | --- |
| EPI497627 | EPI497626 | A/Sapporo/107/2013 |
| EPI497678 | EPI497677 | A/Sapporo/114/2013 |
| EPI497686 | EPI497685 | A/Sapporo/TH1/2013 |
| EPI497680 | EPI497679 | A/Sapporo/116/2013 |
| EPI497682 | EPI497681 | A/Sapporo/119/2013 |
| EPI497684 | EPI497683 | A/Sapporo/120/2013 |
| EPI507761 | EPI507681 | A/Sapporo/29/2014 |
| EPI507764 | EPI507684 | A/Sapporo/33/2014 |
| EPI520521 | EPI520520 | A/Sapporo/83/2014 |
| EPI485787 | EPI485786 | A/Louisiana/08/2013 |
| EPI485779 | EPI485778 | A/Louisiana/07/2013 |
| EPI486652 | EPI486651 | A/Louisiana/10/2013 |
| EPI492813 | EPI492812 | A/Mississippi/11/2013 |
| EPI492805 | EPI492804 | A/Louisiana/13/2013 |
| EPI511899 | EPI508624 | A/Louisiana/01/2014 |
| EPI511898 | EPI516290 | A/Washington/05/2014 |
| EPI517022 | EPI517021 | A/Pennsylvania/14/2014 |
| EPI334772 | EPI334771 | A/Newcastle/2/2011 |
| EPI334770 | EPI334769 | A/Newcastle/17/2011 |
| EPI334774 | EPI334773 | A/Newcastle/37/2011 |
| EPI349160 | EPI349159 | A/Newcastle/125/2011 |
| EPI334776 | EPI334775 | A/Newcastle/53/2011 |
| EPI334780 | EPI334779 | A/Newcastle/82/2011 |
| EPI334778 | EPI334777 | A/Newcastle/62/2011 |
| EPI349115 | EPI349114 | A/Newcastle/86/2011 |
| EPI349163 | EPI349162 | A/Newcastle/129/2011 |
| EPI334784 | EPI334783 | A/Newcastle/89/2011 |
| EPI334782 | EPI334781 | A/Newcastle/85/2011 |
| EPI349158 | EPI349157 | A/Newcastle/102/2011 |
| EPI334766 | EPI334765 | A/Newcastle/132/2011 |
| EPI334768 | EPI334767 | A/Newcastle/151/2011 |
| EPI349201 | EPI349200 | A/Newcastle/168/2011 |
| EPI349297 | EPI349296 | A/Newcastle/179/2011 |
| EPI349299 | EPI349298 | A/Newcastle/212/2011 |
| FJ981613 | GQ377078 | A/California/07/2009 |
| EPI233170 | EPI233169 | A/North Carolina/39/2009 |
| EPI587450 | EPI587449 | A/Jiangxi-Yushui/SWL1220/2014 |
| EPI587452 | EPI587451 | A/Neimenggu-Yuquan/SWL1155/2014 |
| EPI587454 | EPI587453 | A/Hubei-Fancheng/SWL210/2014 |
| EPI587456 | EPI587455 | A/Guizhou-Nanming/SWL1108/2014 |
| EPI587458 | EPI587457 | A/Hunan-Tianyuan/SWL129/2014 |
| EPI587460 | EPI587459 | A/Jilin-Longshan/SWL117/2014 |
| EPI587462 | EPI587461 | A/Heilongjiang-Saertu/SWL1655/2013 |
| EPI587464 | EPI587463 | A/Fujian-Gulou/SWL11609/2013 |
| EPI498558 | EPI498557 | A/Shanghai-Changning/SWL1621/2013 |
| EPI587466 | EPI587465 | A/Chongqing-Banan/SWL1804/2013 |
| EPI587468 | EPI587467 | A/Chongqing-Banan/SWL1732/2013 |
| EPI587470 | EPI587469 | A/Chongqing-Fuling/SWL1638/2013 |
| EPI587472 | EPI587471 | A/Guangxi-Gangbei/SWL1481/2013 |
| EPI587474 | EPI587473 | A/Guangdong-Liwan/SWL1865/2013 |
| EPI587476 | EPI587475 | A/Hunan-Furong/SWL1543/2013 |
| EPI587478 | EPI587477 | A/Heilongjiang-Daoli/SWL1480/2013 |
| EPI587480 | EPI587479 | A/Hubei-Xiangcheng/SWL1750/2013 |
| EPI498564 | EPI498563 | A/Sichuan-Qingyang/SWL1599/2013 |
| EPI587482 | EPI587481 | A/Shanghai-Changning/SWL1621/2013 |
| EPI498567 | EPI498566 | A/Hubei-Wuchang/SWL1322/2013 |
| EPI498561 | EPI498560 | A/Chongqing-Banan/SWL1690/2013 |
| EPI498660 | EPI498659 | A/Sichuan-Wuhou/SWL2259/2013 |
| EPI498657 | EPI498656 | A/Beijing-Xichengnanpian/SWL11619/2013 |
| EPI498606 | EPI498605 | A/Chongqing-Yuzhong/SWL11434/2013 |
| EPI587484 | EPI587483 | A/Heilongjiang-Taoshan/SWL1186/2014 |
| EPI587486 | EPI587485 | A/Yunnan-Xishan/SWL1170/2014 |
| EPI587488 | EPI587487 | A/Shanghai-Putuo/SWL1189/2014 |
| EPI587490 | EPI587489 | A/Fujian-Xiangcheng/SWL2138/2014 |
| EPI587492 | EPI587491 | A/Beijing-Chaoyang/SWL1253/2014 |
| EPI587494 | EPI587493 | A/Shanghai-Putuo/SWL1107/2014 |
| EPI587496 | EPI587495 | A/Jilin-Dongchang/SWL11/2014 |
| EPI498818 | EPI498817 | A/Shaanxi-Hancheng/SWL310/2013 |
| EPI587498 | EPI587497 | A/Sichuan-Dongqu/SWL1871/2013 |
| EPI498777 | EPI498776 | A/Guizhou-Nanming/SWL11969/2013 |
| EPI498639 | EPI498638 | A/Chongqing-Yuzhong/SWL11676/2013 |
| EPI498588 | EPI498587 | A/Jiangsu-Tinghu/SWL1623/2013 |
| EPI498594 | EPI498593 | A/Fujian-Licheng/SWL1527/2013 |
| EPI498600 | EPI498599 | A/Zhejiang-Shangcheng/SWL1623/2013 |
| EPI498603 | EPI498602 | A/Jiangsu-Hailing/SWL11340/2013 |
